# Models of gene gain and gene loss for probabilistic reconstruction of gene content in the last universal common ancestor of life

**DOI:** 10.1186/1745-6150-8-32

**Published:** 2013-12-19

**Authors:** Lavanya Kannan, Hua Li, Boris Rubinstein, Arcady Mushegian

**Affiliations:** 1 Department of Invertebrate Zoology, American Museum of Natural History, New York, NY 10024, USA; 2 Stowers Institute for Medical Research, Kansas City, Missouri 64110, USA; 3 Department of Microbiology, Immunology and Molecular Genetics, University of Kansas Medical Center, Kansas City, Kansas 66160, USA; 4 Present address: Division of Molecular and Cellular Biosciences, National Science Foundation, Arlington, VA 22230, USA

## Abstract

**Background:**

The problem of probabilistic inference of gene content in the last common ancestor of several extant species with completely sequenced genomes is: for each gene that is conserved in all or some of the genomes, assign the probability that its ancestral gene was present in the genome of their last common ancestor.

**Results:**

We have developed a family of models of gene gain and gene loss in evolution, and applied the maximum-likelihood approach that uses phylogenetic tree of prokaryotes and the record of orthologous relationships between their genes to infer the gene content of LUCA, the Last Universal Common Ancestor of all currently living cellular organisms. The crucial parameter, the ratio of gene losses and gene gains, was estimated from the data and was higher in models that take account of the number of in-paralogs in genomes than in models that treat gene presences and absences as a binary trait.

**Conclusion:**

While the numbers of genes that are placed confidently into LUCA are similar in the ML methods and in previously published methods that use various parsimony-based approaches, the identities of genes themselves are different. Most of the models of either kind treat the genes found in many existing genomes in a similar way, assigning to them high probabilities of being ancestral (“high ancestrality”). The ML models are more likely than others to assign high ancestrality to the genes that are relatively rare in the present-day genomes.

**Reviewers:**

This article was reviewed by Martijn A Huynen, Toni Gabaldón and Fyodor Kondrashov.

## Background

The inference of the Last Universal Common Ancestor (LUCA) of all modern cellular organisms can be approached in two ways. The “forward in time” approach uses the knowledge about conditions on the prebiotic Earth, tries to understand what kinds of replicating systems could emerge under these conditions, and proposes the mechanisms for these genetic systems to evolve into LUCA. The “backward in time” approach uses the information about currently living organisms – in particular, about completely sequenced genomes of Bacteria, Archaea, Eukarya, and even viruses – to reconstruct the traits of LUCA. The latter class of methods takes us directly to the last common ancestor of the currently living life forms, rather than to an ancestor of such ancestor [1], and the approach taken here is of that kind.

The problem of *inference of ancestral gene content* has been stated as follows: for each gene in every sequenced genome, determine its state as either ancestral, i.e., present in LUCA, or non-ancestral, i.e., absent from LUCA [1-4]. Since the task is prohibitively difficult for a gene that is found in just one genome, a practical modification of the problem is to label *each set of orthologous genes*, shared by several genomes, as either ancestral or non-ancestral (see [5] for definition of orthology and discussion of issues in practical detection of orthologs). In this study, we suggest a statistical approach to address this problem. We utilize two kinds of data: (a) the evolutionary history of a set of species, modeled as a species’ phylogenetic tree, the root of which is assumed to be the LUCA; and (b) the record of presence and absence of orthologous genes in the same set of species, summarized as phyletic vectors, in which each coordinate represents the status of a gene in one species. As we argue in the last section of this paper, such a framework is a necessary prerequisite to more complex and realistic models of evolution, in particular those that would give the explicit account of horizontal gene transfer between species.

In the context of our current inference problem, there are two classes of evolutionary events that occur along the branches of a tree: gene gain, in which the state of gene changes from absence to presence (in the simplest binary coding of presences and absences, gene gain is depicted as change of state 0→1, and gene loss as 1→0). Any inference of the ancestral state of a gene relies on a quantitative model of such changes.

Different methods for ancestral state reconstruction, including maximum parsimony (MP) [2,6,7] and approaches based on more extensive modeling, such as maximum likelihood (ML) and Bayesian inference, have been introduced (e.g., [8]). The MP approach infers the ancestral states by starting with the current states of each gene at the tips of the tree and proceeding backwards in time, to the root, minimizing the total number of events (gains and losses) during the evolutionary history of a given set of species. As always with parsimony approaches, it is possible that two or more scenarios consist of different events but have the same (minimal) number of them; this requires additional criteria for breaking the ties. More important, it is not clear that unweighted parsimony, which in effect postulates that a gain and a loss of a gene are equally likely, is best compatible with the data. Mirkin *et al.*[2] proposed the weighted parsimony approach, which takes into account the possible difference between gene gain rate and gene loss rate. This was done by using a parameter called gene penalty, defined as the ratio of gene gain rate to gene loss rate. It was observed, however, that the ancestral gene sets constructed with the gain penalty *g*=1 tended to have the smallest number of genes whose predicted functions were biochemically coherent enough to sustain life, suggesting that the number of gene gains and losses encountered by a system may be at approximate equilibrium.

Methods based on maximum likelihood are of interest because they can take into account more information about the process of gene gains and gene losses, and because they can reflect the uncertainties in deciding the state of the gene at each ancestral node in the tree by assigning probabilities of presence and absence of each gene at this node. The likelihood framework can also incorporate the knowledge of branch lengths in the species tree and the lineage-specific differences between the frequencies of various classes of events across different genes.

Likelihood-based reconstruction of ancestral molecular traits have been attempted in the recent years (see [9-13]), focusing mostly on inferring the ancestral nucleotide or protein sequences on the basis of sequences from present-day species. These approaches model the evolutionary history of an orthologous nucleotide or amino acid site as a continuous-time Markov process, in which the substitution rates are associated with time (tree branch length) and are estimated by maximizing the likelihood of the given phylogenetic tree and the sequences of a specific gene of interest. The most likely ancestral state of each site is then chosen by evaluating the marginal probability for each state. Many of these models can be modified to deal with the ancestral gene content problem.

Cohen *et al.*[8] have used a likelihood framework to analyze the binary gene presence-absence vectors for multiple orthologous genes in a set of existing species with completely sequenced genomes. Their analysis allowed the gene gain and loss rates to be unequal, and the results indicated that the gain and loss rates that vary between different gene families explain the observed data better than the constant gain and loss rates. In another study, presences and absences were replaced with multiple states for the gene family size, to describe the history of a gene in relation to duplications and gene losses in the MP framework, without explicitly reconstructing gene content in LUCA [7].

Here we extend this class of models to examine the changes between the states of gene absence, of a single-copy gene presence, and presence of a group of in-paralogs, in the maximum likelihood framework. The calculation of the probability of the ancestral presence (“ancestrality”) of each gene uses the information on the changes in the number of in-paralogs of a gene in evolution. We explore several likelihood models of increasing complexity. Our results indicate that, when more than two states of genes are allowed, the estimated gene loss rates tend to be higher than estimated gene gain rates, with the loss-to-gain rate ratios around 6 for the majority of COGs. All models give relatively close estimates for the number of genes in LUCA, around 500 genes, but the identities of genes that are confidently placed into LUCA are different under different models. Probabilistic approach of that kind is a necessary step towards more detailed, quantitative reconstructions of gene content and metabolic networks in LUCA.

## Results

### Probability of state transitions along a branch

The probabilistic models can be used to infer whether there has been a change in the gene family size between the ancestor and the descendant along each branch in the species tree. This is done by substituting the rate parameters that optimize the likelihood function in the transition probability matrix *P*(*t*) (refer to the Methods section for the definitions), where *t* is the length of the branch. Using these transition probabilities, the probabilities of each state at LUCA can be calculated. Each of the models discussed in this work suggests that, even as gene losses and gene gains occur in evolution (the off-diagonal entries in the transition probability matrix), the most likely outcome along any branch is that the gene family size remains the same, with higher probabilities for maintaining gene absence than for maintaining gene presence. Another common property of all models (with the exception of model (B1), which is constrained to have the same rates of gene gain and gene loss) is that gene losses are typically from two to four times as likely as gene gains. The median transition probability matrices (with the highest probability in each row highlighted) for a branch with the length 0.35 (the median of the observed branch lengths in the tree) are

Additionally, transition probabilities of models (M1) and (M2) suggest that the state of multiple in-paralogs is more prone to changes along a branch than the state of a single-copy gene. The second rows of these probability matrices indicate that acquiring a new gene is less likely than duplicating the existing gene in the species, and that the loss of an existing gene is more likely than its duplication. The main difference between the models (M1) and (M2) is in the gene loss transition probabilities when there are multiple copies in the ancestor. In model (M2), it is less likely that a gene loses all its copies along a branch, whereas in (M1) the probability of losing all copies of genes along a branch is about the same as the probability of maintaining multiple copies of the gene.

### The ancestral probabilities

For each model discussed in the previous section, the probability that each COG appeared in LUCA can be inferred. A gene set LUCA-ML*x* consists of genes whose ancestral probabilities are at least *x* in their preferred model among (M1) and (M2). Table 1 (column II) shows the number of gene sets that are inferred as ancestral under the different values of *x* from 0.5 to 1. We construct an ancestral COG list using the probability 0.7; whenever the probability level is not stated, we refer to LUCA-ML 0.7 as LUCA-ML.

**Table 1:** Number of ancestral COGs included in LUCA-Mlx for various probability values for x

**I**	**II**	**III**	**IV**	**V**
0.5	1155	716	439	78
0.55	890	477	413	104
0.6	783	389	394	123
0.65	667	304	363	154
0.7	597	251	346	171
0.75	509	195	314	203
0.8	443	154	289	228
0.85	372	105	267	250
0.9	319	73	246	271
0.95	267	46	221	296
1	47	0	47	470

ML*x*, where the parameter *x* is the smallest ancestrality that the COG must have to be included into LUCA. The numbers of COGs in different ML*x* and their overlap with LUCA 1.0 [2] are also shown. Column I - *x*; Column II - number of COGs in LUCA-ML*x*; III - Number of COGs found in LUCA-ML*x*, but not found in LUCA 1.0; IV - Number of COGs found in both LUCA-ML*x* and LUCA 1.0; V - Number of COGs found in LUCA 1.0, but not found in LUCA-ML*x*.

Our LUCA-ML is not the same as LUCA1.0 reconstructed in [2], most likely because the two ancestors were inferred using different methods, which were moreover applied to different sets of species and COGs. LUCA-ML 0.7 and LUCA-ML 0.6 share, respectively, about 57% and 50% of their genes with LUCA 1.0, and more than 65% of LUCA 1.0 are included in each of our ML ancestral gene sets.

### Gene content of LUCA-ML 0.7 and LUCA-1.0

The proportion of all COGs that is scored as ancestral is similar in the two reconstructed ancestors - 23% of total in the case of LUCA 1.0 (517 COGs) compared to 26% (597 COGs) in LUCA-ML 0.7. On the other hand, the identity of the COGs in the two sets differs considerably, with only 346 COGs found in both sets.

**Figure 1:**
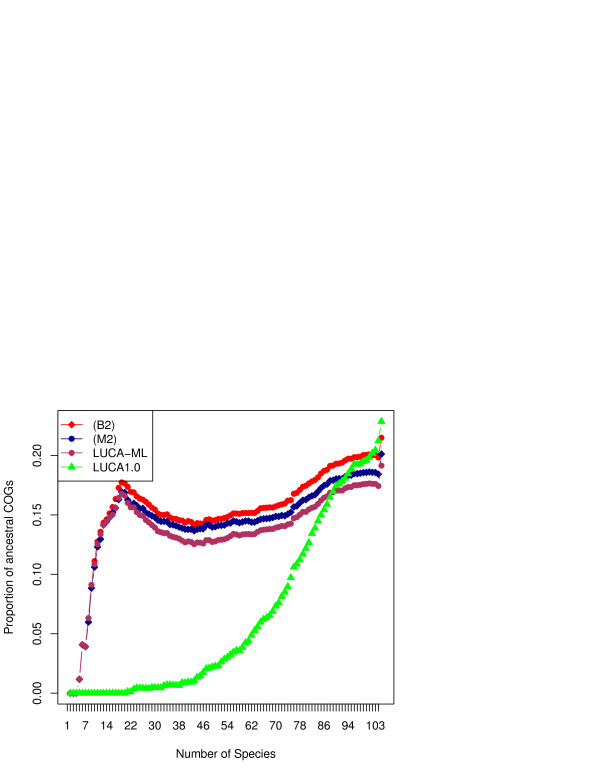
Distribution of all COGs under models B2 and M2, as well as high-ancestrality COGs (LUCA-ML and LUCA1.0), by the number of genomes in which they are present.

Figure 1 shows the distribution of input set of COGs as well as inferred ancestral sets by the number of genomes in which they are found under different models. The number of COGs in LUCA 1.0 and LUCA-ML 0.7 are similar for those COGs that are found in more than 80 genomes, but differ considerably for rare COGs; model (M2) and other ML approaches tend to place higher proportion of sparsely distributed COGs into LUCA.

High-level classification of the known and predicted molecular functions of the ancestral COGs is shown in Table 2.

**Table 2:** Distribution of COGs based on their functions

**I**	**II**	**III**	**IV**	**V**
A	1	0	0	0
B	1	0	0	1
C	179	20	26	24
D	16	6	2	1
E	183	**67**	19	16
F	70	31	12	6
G	99	13	6	6
H	112	24	18	16
I	48	10	7	3
J	104	**64**	12	10
K	65	10	7	10
L	123	16	12	22
M	75	9	1	4
N	13	1	0	1
O	81	12	13	5
P	113	16	9	10
Q	25	1	1	1
R	297	29	16	**49**
S	343	6	4	**55**
T	37	2	6	5
U	15	4	0	3
V	24	5	0	2
X	237	0	0	1

Column I - Broad functional categories according to the COG resource; Column II - Total number of COGs in each category; Column III - Number of COGs in both LUCA 1.0 and LUCA-ML 0.7; Column IV - Number of COGs found in LUCA1.0, but not found in LUCA-ML 0.7; Column V - Number of COGs found in LUCA-ML 0.7, but not in LUCA 1.0.

Poorly characterized conserved genes (categories R and S) are more frequent among the COGs that were scored as ancestral by the ML approach only, which correlates with higher proportion of rare COGs in these categories and relative favoring of these COGs by the ML approaches. These “high-ancestrality” COGs from the R and S categories account for about 16% of all COGs in these functional groups, and more insight into their function will be useful for better understanding of ancestral biochemistry.

The other extreme in “ancestrality” is represented by the COGs that belong to the category J (Translation Machinery and Ribosome Biogenesis), as well as category E (Amino Acid Biosynthesis). The vast majority of all COGs in these two categories were predicted to be ancestral by all approaches, which may be attributed in large part to their broad distribution in the genomes.

**Figure 2:**
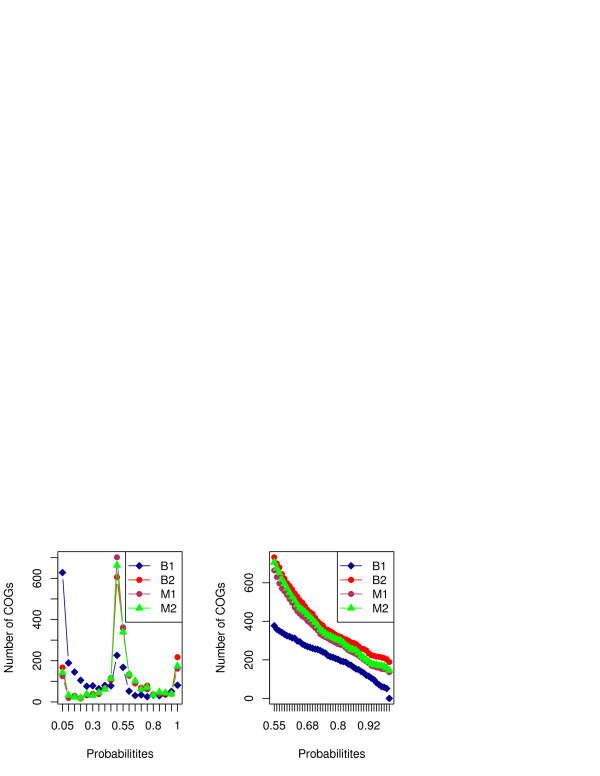
**Probability distribution of the COG ancestrality under various models.** The first panel shows the frequency of COGs with the different probability of occurrence at LUCA, and the second panel shows the number of COGs above the different probability thresholds.

Figure 2 shows the distribution of all COGs by probability of being ancestral under each model, as well as the number of ancestral COGs under different probability cutoffs. The probabilities are well distributed throughout the range, but a considerable fraction of them (at least 15%) are clustered around 0.5. This is the “gray zone” of ancestrality, which may be resolved by future analysis, some directions of which are discussed below.

## Methods

### Data and inputs

#### COG dataset

A publicly available release of the NCBI COG dataset [14], consisting of 14714 COGs and representing 346378 proteins in 87 bacterial and 16 archaeal genomes, was used in this work. Eukarya are generally (even if not universally) considered to be derived life forms, likely to have arisen from a merger of a bacterium and an archaeon [15-17], so we did not use their gene content in this study. Among the 14714 COGs, 1795 are found only in Archaea and 10658 are found only in Bacteria; if the root of the species tree is conventionally placed between Bacteria and Archaea, these genes are unlikely to be included into LUCA with a probability higher than 50%. We did not consider these, Bacteria-only and Archaea-only, classes of COGs in our present analysis. Among the remaining 2261 COGs, 47 appear in all genomes and would be reconstructed as ancestral under any model, unless horizontal gene transfer is taken into account (see Discussion and conclusions). The remaining 2214 COGs are found in archaeal as well as in bacterial genomes and include 185257 genes. Just 421 COGs contain exactly one ortholog per genome. In contrast, 1793 COGs (81%) have in-paralogs, i.e., a group of genes more closely related to each other than to any homolog in another species, in at least some genomes; on average, there are about 6 paralogs per COG, and if there are paralogs in a COG, they are found on average in 12 species.

#### Species tree

**Figure 3:**
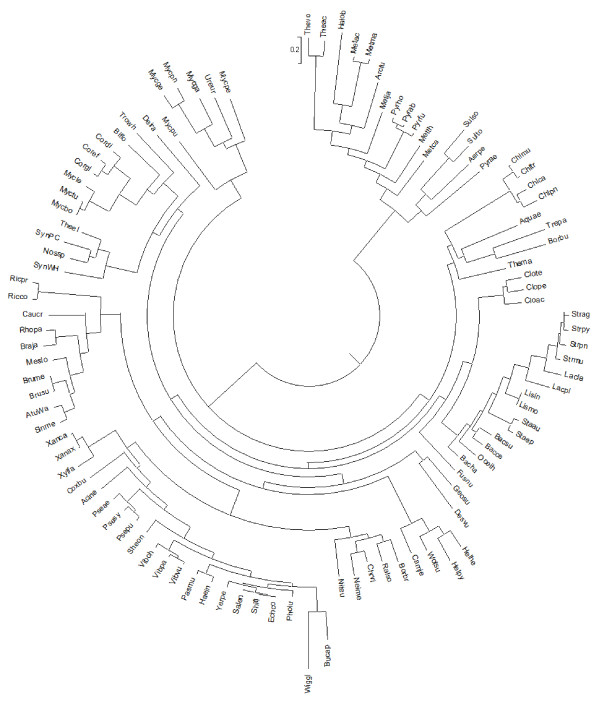
**The reference species tree.** The reference species tree was constructed using the sequences of 11 universal genes COG00081, COG00093, COG00096, COG00097, COG00099, COG00102, COG00103, COG00197, COG00244, COG00256, COG00533.

The reference species tree (see Figure 3) was constructed by concatenating the sequence alignments of 11 COGs that are present in all 103 genomes, have no duplications and no evidence of horizontal gene transfer [18]; ten of them are ribosomal proteins and one is a tRNA modification enzyme. The super-alignments were used to build the consensus tree with 1000 bootstrap replicates using PROML (maximum-likelihood module) in the PHYLIP package [19]. This reference species tree has its canonically defined root (representing the LUCA) between Bacteria and Archaea.

#### Phyletic vectors

For each gene in each genome, one of the states 0, 1 or *m* is assigned, depending on whether there are 0, 1 or multiple copies (in-paralogs) of the gene in this genome. The list of 0, 1 and *m*, ordered by species and representing the known state of gene/COG in the present-day genomes is called *phyletic 0, 1, m-vector*. In the case when there are multiple copies of a gene in a species, the gene gain process can be modeled either as a single event 0→m, or as a sequence of several events, for example 0→1 event (gene gain in a strict sense) and 1→m (gene duplication, lineage-specific expansion, or gain of gene copies) event. Likewise, gene losses can be modeled either as a single event m→0, or as two classes of events, those of complete gene deletion (1→0 gene loss) and lineage-specific in-paralog loss (m→1). Probabilities of those different events may be different. Indeed, it stands to reason that it may be easier to lose a single-copy gene than a whole group of in-paralogs, and it may be easier to increase the number of in-paralogs of an already-existing gene than to acquire the first copy. (Note that the model does not utilize the instantaneous rate of a molecular gain or loss event in an individual genome, but rather the rate of fixation of the new gene state in the population, and we always use “gene gain” and “gene loss” in this latter sense). This agrees with the results showing that the distribution of gene numbers within gene families and orthologous groups often can be approximated by a power law [20-24]. More recently, it has been shown that the rate of any gene-count change in protein families is directly proportional to the family size [25], thus supporting the Markovian nature of the process. In this paper, we reconstruct the ancestral gene content using the likelihood model by incorporating the rate heterogeneities. We do not model the horizontal transfer events separately from other gene gains, but in the Discussion and conclusions section we discuss how these transitions may be incorporated in the likelihood method in the future work.

### Substitution-rate matrix and transition-probability matrices

For a given phyletic 0,1,*m*-vector, we propose a family of ML models that employ different sets of parameters. The model can be specified by giving its *substitution-rate matrixQ*=(*q*_*i* *j*_), which is a 3×3 matrix, whose rows represent the ancestral states and the columns represent the descendant states, the off-diagonal entries *q*_*i* *j*_ (*i*≠*j*) represent the instantaneous rate of fixation of state change from state *i* into state *j* in the species, and the diagonal elements *q*_*i* *i*_ are chosen in such a way that the row sums are 0. The matrices are given by

where the transition rates *g*_*i*_, *l*_*i*_, *c*_*i*_, *i*=1,2 are the unknown parameters and for the model (M1) *c*_1_= *c*_2_=*c*.

We compare these models with simpler models defined on binary phyletic vectors, such as the substitution matrices described by Cohen *et al.*[8]. We call these earlier models (B1) and (B2), and their 2×2 substitution-matrices are respectively

where *g*, *l* are unknown transition rates that can be estimated and for the model (B1) *g*=*l*=*c*.

The models developed in [8] and our models (B1) and (B2) are identical in the form, but they generally speaking give different rate estimates because they were derived from different datasets (we used a version of the COG resource that has more genomes and more genes).

Using the substitution-rate matrices, the probabilities of transition between states along a branch with length *t* can be calculated. The set of probabilities is represented by a matrix *P*(*t*) called the *transition-probability matrix*, whose *ij*^*t* *h*^ entry represents the probability of transition from state *i* to state *j* along the branch with length *t*, measured in the time units. With the assumption of continuous Markov chains (*i.e.*, *P*(*t*_1_+*t*_2_)=*P*(*t*_1_)*P*(*t*_2_) for any two time intervals *t*_1_ and *t*_2_), we have *P*(*t*)=*exp*(*Qt*)=*I*+*Qt*+(*Qt*)^2^+⋯, with the infinite sum of the matrices always converging (refer to [11, Chapter 1] for details). Expressions for the models (B1) and (B2) have been given in [8].

### Likelihood reconstruction and ancestral state inference

**Figure 4:**
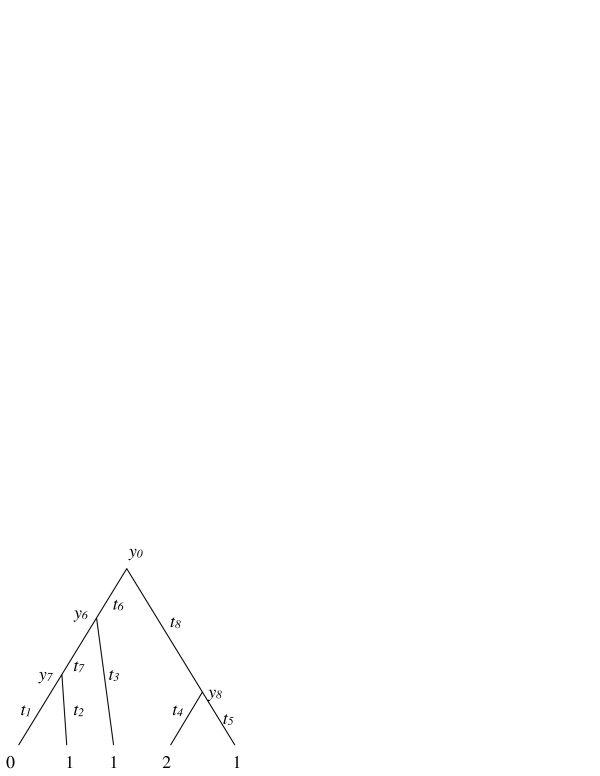
**A toy example showing the parameters for calculation of the likelihood function.** The elements of the phyletic vector of a gene are shown at the leaves. Branch lengths *t*_1_, *t*_2_, ⋯, *t*_8_ are known.

Given the species tree and the phyletic 0, 1, *m*-vector of a gene/COG, we want to find the most likely ancestral state of the gene described by the vector under the given model. The ML framework allows us to assign a probability for each state of the gene at each node in the tree, moving from the states in the present-day genomes (leaves of the tree) in the direction of the root. The probability of a state at each ancestral node depends on the probabilities of this state at its two descendants. Figure 4 shows an illustrative example of a tree with five extant species, the states of a particular gene in these five species given by the phyletic vector *X*=(0,1,1,2,1). Let *t*_1_, *t*_2_, ⋯, *t*_8_ be the known branch lengths (estimated simultaneously with constructing the species tree), *y*_6_, *y*_7_ and *y*_8_ be the unknown gene family sizes of the three internal ancestral nodes of the tree, and let *y*_0_ be the gene family size at the LUCA, each of *y*_*i*_ taking values from {0,1,*m*}. Consider model (M1) with the parameter space of substitution rates between states, $\theta =\left(g_{1},g_{2},l_{1},l_{2},c\right)$, and let *Y* be the unknown vector (*y*_0_,*y*_6_,*y*_7_,*y*_8_). The likelihood, *f*(*X*|*θ*), of observing the data *X* is given in terms of all combinations of possible states for *y*_0_, *y*_6_, *y*_7_ and *y*_8_, as

$$\;\begin{array}{l} f(X|\theta )\;=\underset{Y}{\sum }f(X|Y,\theta )f(Y)\\ \;=\underset{y_{0}}{\sum }\;\underset{y_{6}}{\sum }\;\underset{y_{7}}{\sum }\underset{y_{8}}{\sum }\;\left[\pi_{y_{0}}p_{y_{0}y_{6}}\;\left(t_{6}\right)p_{y_{6}y_{7}}\;\left(t_{7}\right)p_{y_{7}0}\left(t_{1}\right)p_{y_{7}1}\right)\\ \;\times \left(\left(t_{2}\right)p_{y_{6}1}\left(t_{3}\right)p_{y_{0}y_{8}}\left(t_{8}\right)p_{y_{8}2}\left(t_{4}\right)p_{y_{8}1}\left(t_{5}\right)\right], \end{array}$$

where *f*(*Y*) represents the prior probability of *Y*, and *f*(*X*|*Y*,*θ*) is the conditional probability of observing *X* given *Y* and *θ*. Also, $\pi_{y_{0}}$ is the prior probability of the gene family size at the root (i.e., the probability that *y*_0_=*k*, where *k*∈{0,1,*m*}) and *p*_*i* *j*_(*t*) for *i*,*j* ∈{1,2,*m*} are the elements from the transition probability matrix *P*(*t*). The likelihood is simplified as

(1) $$f(X|\theta )=\underset{y_{0}}{\sum }\pi_{y_{0}}L_{0}\left(y_{0}\right),$$

where $L_{0}\left(y_{0}\right)$ is the likelihood of observing *X*, given that the gene family size at the root is *y*_0_[11, Chapter 4]. To reconstruct the gene family size of the last universal common ancestor, the root node *y*_0_, we calculate

(2) $$f\left(y_{0}=k|X,\theta \right)=\frac{\pi_{y_{0}=k}L_{0}\left(y_{0}\right)}{f(X|\theta )}.$$

The probabilities for each of the states *k*∈{0,1,*m*} are calculated, and the state with the highest probability is assigned to the LUCA. The parameters in the *Q* matrix are estimated by maximizing the likelihood function given in Equation (1). Then, the probability density function for *y*_0_ (probabilities of ancestral states at LUCA) is calculated using the Equation (2).

### Prior probabilities

In this study, we are interested in inferring the presences and absences of ancestors of the present-day genes in LUCA. The states at the LUCA are, most likely, 0 or 1, but there is also a possibility that some of the genes had closely related paralogs already in LUCA. We assigned the following prior probabilities $\pi_{y_{0}}$:

$$\pi_{y_{0}=0}=0.5,\pi_{y_{0}=1}=0.45\;\text{and}\;\pi_{y_{0}=m}=0.05.$$

The values of the priors for presence is in the ratio of 9:1 for single gene versus at least two in-paralogs at LUCA, i.e., the proportion of ancestral COGs with in-paralogs is several times smaller than for the extant COGs set (81% of COGs that we considered have in-paralogs). This reflects our sense that LUCA, though not necessarily of a minimal size, is more likely to have had a relatively small rather than a very large genome. Note that these assumptions are distinct from the knowledge that LUCA definitely included a collection of very ancient paralogs – for example, the main catalytic domains of aminoacyl-tRNA synthetases that belong to only two monophyletic sequence families [26]; the paralogs of this kind are represented by distinct COGs.

Additionally, we weight the prior probabilities with the frequency of occurrence of each gene across the present-day genomes. For example, if a COG is found in 87 current genomes out of 103, a prior probability of 87/103=.84 is multiplied by the probability of the presence at LUCA, and the product is scaled appropriately, such that the probabilities of presence and absence sums to 1. This becomes necessary especially for genes found in nearly-all species, for which the stochastic nature of the Markov process occasionally results in a implausible inference of absence at the LUCA. Multiplying by the frequencies of occurrences corrects this problem (data not shown).

### Model comparison

For each COG, the model that is the best fit to its phyletic vector can be found by calculating the Akaike Information Criterion (AIC) [27], which is

AIC=−2l+2p,

where *l* is the optimum log likelihood under the model, and *p* is the number of parameters. In models (M1) and (M2), we have *p*=5 and *p*=6 respectively, and for models (B1) and (B2), we have *p*=1 and *p*=2 respectively. The AIC criterion can be applied only to compare models that use the same datasets and hence we use the criterion to decide for each gene, which model among (M1) and (M2) (or among (B1) and (B2)) it prefers. The R code implementing the models and the estimation of the maximum likelihood parameters is available at https://github.com/lavkan/LUCA.

## Discussion and conclusions

In this work, we proposed the maximum likelihood-based models, which use the consensus phylogeny and the states of genes (absence, presence and copy number) at the leaves of the phylogenetic tree to infer the status of each gene in the common ancestor of all examined species - in this case, the Last Universal Common Ancestor of living organisms. Perhaps the main general conclusion from this work is that models with more parameters, i.e., those in which the rates of transition between various gene states are estimated separately, are more likely to place rare genes in the common ancestor, provided that these genes are found in different clades.

Despite more detail of the evolutionary process embodied in our models than in simpler parsimony-based models, we do not feel yet that the results accurately represent the hypothetical organism of the ancient past. The detailed analysis of the biological functions of genes that “make it” into LUCA must await several further improvements. Some of such future directions are outlined presently.

First, our current models assume that the rates of gene gain and gene loss are constant across all branches of the phylogenetic tree for each COG. This assumption is clearly a simplification, and future models should include rate heterogeneity, for example, as delineated in [28]. It would be interesting to see which effect these modifications have on the ancestral gene counts.

Second, better theory may be applied to the choice of priors, apart from just using the frequency of gene presence in the current genomes.

Third, if a gene does not belong to any orthologous group, nothing can be said about its ancestrality. This problem of lost genes can be partly corrected in several ways. Likelihood correction for missing data (See references [15] and [16]) may be employed to improve the estimate of the number of genes in LUCA, though of course it will not directly reveal the identity of the lost genes. It should be noted also that a COG by construction includes at least three genes, one in each of three taxonomically diverse lineages [29], but there are also pairs of genes, “pre-COGs”, that have the potential to form COGs when new genomes are added to the dataset; studying the “pre-COGs”, in particular those found in two very distant species, may provide additional clues to the ancestral gene repertoire.

Fourth, there is the gray zone of ancestrality, which contains a large proportion of all COGs (Figure 2). It is worth trying to resolve the contents of this zone better. Several approaches can be envisioned here; perhaps most useful among them would be to include the analysis of the biological coherence of the inferred ancestral COG set (see [30] for similar considerations at a more recent evolutionary scale). For example, if many genes that belong to a known biosynthetic or signaling pathway have high ancestrality, but one or a few are in the “gray zone”, the additional evidence of functional linkage and tendency for co-inheritance may be used to move the latter group of genes into LUCA.

Fifth, broader and denser sampling of genomes should improve the accuracy of the inference, by adding more COGs to the dataset and by discovering new members of already existing COGs.

**Figure 5:**
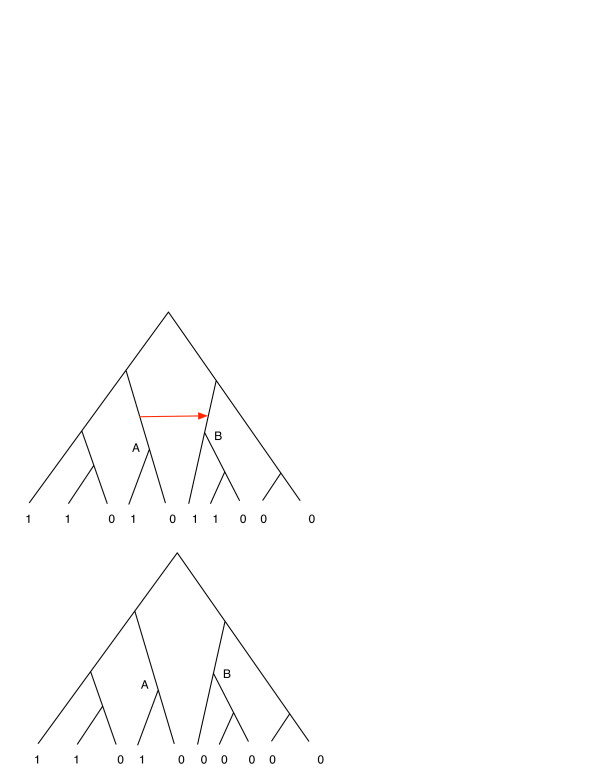
**The strategy of editing phyletic vectors on the basis of information about HGT.** A horizontal gene transfer event (red arrow) from ancestral species A to species B is inferred on the basis of comparison of the topologies of the species tree and the sequence family tree (not shown in this figure). The bottom vector is obtained by replacing all the presences by absences in all descendants of B, and the inference of ancestral state can be updated on this edited vector.

These modifications to our approach, however useful, still set to one side the problem of the uncertainty of the species tree topology. In fact, the foundational idea that the evolution of the living forms on Earth is properly represented by a strictly bifurcating, cycle-free graph known as the Tree of Life is itself under re-evaluation, primarily because of considerable evidence of horizontal gene transfer, which occurs in the present-day species [31,32] and must have been even more prevalent at the earlier stages of evolution [33,34]. Radical proposals to abandon the tree-like phylogenetic representations have been made [35,36]. If taken to the extreme, the idea of rampant ancient horizontal gene transfer suggests that all bets are off in phylogenetic inference at a long evolutionary distance: large portions of evolutionary history may be completely erased by HGT [37]. On the other hand, taking account of the HGT events, for example by comparing the species tree to the sequence alignment-based tree of each protein family [38,39] may help to refine the inference of gene content in LUCA. Figure 5 illustrates a tentative strategy for such a refinement: if a gene can be shown to have been horizontally transferred from the left-hand clade in the tree to the right-hand clade, all coordinates of the phyletic vector of this gene corresponding to the species within that right-hand clade have to be reset to zero, and the ancestral state inference has to be done on the edited vector. The net effect of this approach will be to reduce the number of genes in LUCA.

These developments will result in a more accurate inference of gene content in LUCA, which can be tested for functional coherency and perhaps even submitted, in the not-too-distant future, to synthetic biologists for practical reconstruction.

## Reviewers’ comments

*General note from the authors:* We thank Dr. Huynen, Dr. Gabaldón and Dr. Kondrashov for reviewing our manuscript. All three reviewers are wondering about the computational experiments that we are proposing to do but have not done ourselves. Unfortunately, none of the authors are in a position to continue work on this project at the moment. On the other hand, we see no upside in keeping quiet about the work that has been done already, as well as about the future directions that we or others could take with the models that we have developed. We also realized that the oroginal title of the manuscript, “Probabilistic reconstruction of gene content in the Last Universal Common Ancestor of Life” was claiming more than was actually delivered, and therefore changed it to “Models of gene gain and loss for probabilistic reconstruction of gene content in the Last Universal Common Ancestor of Life”.

### Reviewer 1: Martijn A Huynen, Nijmegen, Netherlands

The manuscript by Kannan et al, extends a probabilistic framework for ancestral genome content reconstruction to also include varying numbers of genes per orthologous group per genome. This extension is valuable, the manuscript is well written, the methods are well explained and the main conclusion about the presence in LUCA of sparsely distributed COGs appears justified. In general I would have liked to see however either a more thorough analysis of the performance of their model under varying assumptions or datasets. I specifically wonder how the number of included species affects the results, and how including e.g. more Archaeal genomes (e.g. from the ‘TACK superphylum’) affect LUCA.

Authors’ response: We agree that the breadth and density of taxon sampling are important and should be improved. This will positively impact most aspects of the model, as well as produce more orthologous groups to work with.

Alternatively I would have liked an expansion of the methods to also include Horizontal Gene Transfer. It is nice to propose a strategy, but why then not implement it and study its effects? I do find the current manuscript lacking in sufficient novelty of methods & results. Furthermore some aspects of the results are only intelligible for people who work with COGs on a daily basis.

Authors’ response: We tried to improve the presentation by taking into account specific comments as well as editing the manuscript again.

More specific questions: I am not convinced by the argument: “The values of the priors for presence is in the ratio of 9:1 for single gene versus two in-paralogs at LUCA”.

Authors’ response: Admittedly, as with many other aspects of the model, it would be better to derive the prior probabilities, perhaps even gene-specific ones, from the data. This is for the future. The statement in the manuscript about “two in-paralogs” is now changed to “at least two”, consistent with *y*_0_=*m* index.

Why would LUCA be smaller than current genomes? What happens when that ratio is decreased?

Authors’ response: The assumption that ∼10% of genes in LUCA have in-paralogs, obviously, adds >10% to its gene count. In our experiments, LUCA tends to be on the smaller side but within the range of current genomes of extracellularly living parasitic bacteria. More accurate count awaits the improvements that we discuss in the paper, including the resolution of the “gray zone”, i.e., COGs with ancestrality close to 0.5.

It would be nice to get an intuitive explanation why “model (M2) and other ML approaches tend to place higher proportion of sparsely distributed COGs” in LUCA.

Authors’ response: We think that this is because our models capture the reality better, accounting not only for the number of splits in the tree (as parsimony approaches, especially the unweighted ones, essentially do) but also for branch lengths and for repeated gains/losses.

Why is there such a weird “shoulder” on the left side of the Figure 1: i.e. why is the fraction of ancestral COGs higher for COGs that occur in 14 species than for COGs that occur in 40 species?

Authors’ response: This may be traced back to the uneven sampling of species in the tree. Te 40-species COGs are enriched in proteobacteria-specific genes (there are 48 species of proteobacteria in the dataset) and are more often placed not earlier than the root of that clade. The 14-species COGs tend to include orthologs sparcely distributed in bacteria as well as archaea and have relatively high ancestrality.

Can the phylogeny in Figure 3 be rooted in the way it is used in the manuscript? It might be nice to mark the various taxa (Archaea etc.). Where can one find what the species abbreviations mean?

Authors’ response: In the initially submitted image, the root was misplaced; this has been corrected now. Species abbreviations can be found in ftp://ftp.ncbi.nih.gov/pub/wolf/COGs/COG0508/genomes.CSV.

What does the second panel of Figure 2 signify?

Authors’ response: Explanation provided.

In general the legends with the figures are very short. I take it e.g. that Figure two refers to the estimated number of COGs in LUCA? It would be nice to be able to understand the meaning of the figure from its legends, now one has to search in the manuscript. Also a Legend of Table 2 that says: “Column I - Letters;” may be intelligible to the people who work with COGs on a daily basis, but not to people who this manuscript might hope to reach.

Authors’ response: We provided more detailed legends.

References: Power laws in gene family size distributions in genomes have first been observed by Gerstein (PMID:9417935) and have first been modeled by this referee (PMID:9580988).

Authors’ response: Indeed, we should have included these references in the first place, and we now do. Going even further back, similar trends have been tabulated in PMID: 8524875 (1995) and plotted in PMID: 7477316 (1995), though neither of these efforts commented on the form of the distribution at the time.

Editorial: Page 2: this was done (missing capital) Why is “is” on page 7 underlined?

Authors’ response: Corrected.

### Reviewer 2: Toni Gabaldón, Centre for Genomics Regulation (CRG), Spain

This paper applies different Maximum Likelihood approaches, extensions of previously-proposed models, to the problem of inferring the gene complement in the Last Universal Common Ancestor, and compares the results to previous estimates. The authors rightly recognize the large limitations of their approach, which they discuss to the point of finally assuming that their result do not “accurately represent” LUCA. The enthousiastic reader (I must admit I was one, deeply interested in the issue of early evolution of life) is thus left with a bitter taste, and, most importantly, with the question: what is then the contribution to resolving the composition of LUCA?

Authors’ response: We hope that the contribution is in the probabilistic model itself and in its implications, in the practical code (see note about the availability) and in outlining some directions of the future research program.

Admitedly, the problem at hand is a daunting task and one is ready to accept results that do not “accurately represent” LUCA but which can be considered “reasonable approximations”. The authors do not evaluate how reasonable is their reconstruction other than by comparing it -mostly numerically but not qualitatively- with previous approaches. To really assess whether the new implementations in the probabilistic models are going in the right direction towards reconstructing a more accurate LUCA one would need to look into the properties of the reconstructed genomes. I understand that is difficult to assess how reasonable an ancestral reconstruction is, but previously explored ideas include looking for completeness of pathways (rather than rough categories) inferred to be present ancestrally (e.g. translation, replication, membrane, etc). The implementations developed by the authors seem more realistic than previous assumptions but it would be necessary to test whether their use actually translates into more reasonable reconstructions.

Authors’ response: We agree. Someone will address these questions in the future, perhaps even using the framework contributed in this study.

The authors acknowledge the uncertainty in the prokaryotic tree of life. This includes not only the topology, but also the branch lengths. They used their own reconstructed tree from just 11 conserved COGs. How this differs to other species trees available (e.g. that of Cicarelli et. al. (Science 2005)) is not discussed, but I understand there are topological and branch length differences. Of importance for the discussion at hand is how robust is the LUCA reconstruction to these given parameters (fixed priors, after all). The authors could easily test whether some variations in the species tree (e.g. an alternative topology reconstructed by other authors, or with a subset of the genes, or changing the scale of the branch lengths) dramatically affects the reconstruction.

Authors’ response: There are small differences in the topology and branch lengths between our tree and Cicarelli’s, as they were inferred from the overlapping but different character sets. We would like to emphasize the likelihood models and their comparison in this work (see change in the title and the edits of the manuscript), so the topology of phylogeny is a subject for another day.

Another relevant aspect is the dataset used. It is clear that this is the primary data and an accurate reconstruction will depend on the availability of a sufficiently dense and balanced sampling of extant genomes. To start with they discard ∼80% of the COGs because they are only present in Archaeal or Bacterial genomes, I wonder how many of them would still be “domain-specific” when considering the 7000+ currently available prokaryotic species. COG is a wonderful resource but it is somehow out of date with other databases representing a more complete view of currently-available genomes. For instance EggNOG (http://eggnog.embl.de/version\_3.0/) uses an approach based on COG but it is computed over 1000+ genomes, other resources may be even more comprehensive. Do the authors consider the version of COG as a sufficiently balanced sampling of extant genomes? I think a subsample of similar size from the currently available genomes would be a fairer representation of the diversity of the prokaryotic lineages, specially for archaea. Similarly to the discussion with the tree, a recomputation of LUCA with another genome sampling may have some effect which would be interesting to measure. Altogether I think the presented models are a valuable contribution to the field of reconstructing ancestral proteomes, but with the data presented is difficult to assess whether the resulting models are actually improving our inference on the ancestral common ancestor. I encourage the authors to pursue the goal of qualitatively assessing the reconstruction, this will help them to assess progress with the planned future implementations. Finally, the authors ommit any discussion on the inferred characteristics of the reconstructed LUCA. As mentioned before, some discussion on the processes inferred to be present in the reconstructed LUCA will be a way to assess and compare the different approaches. Moreover the ultimate goal of reconstructing LUCA is precisely to learn something about the biology of ancestral organisms and their possible environment. I wish the authors had presented some discussion in this regard. This would make the paper even more interesting.

Authors’ response: We agree that the last publicly available release of NCBI COGs is outdated and EggNOG would be better in all respects. This is one reason why we stop short of actually describing the gene repertoire of reconstructed LUCA in any detail (the other reason, of course, is that neither we nor others have developed a fully satisfactory way to account for the false positives due to horizontal gene transfer events - see text).

Minor comments The authors implement their model in R code “available upon request”. It would be recommended to directly provide this through a public repository of code, such as github or others.

Authors’ response: Done.

In the introduction the authors discuss some previously developed methods for the reconstruction of ancestral genomes. I missed some mention to alternative approaches that are based on the analysis of gene trees rather than on phyletic profiles, although they have beed used for more recent ancestors (e.g. Gabaldón, T. and Huynen, MA. (2003) Reconstruction of the protomitochondrial metabolism. Science 1;301(5633):609.).

Authors’ response: Cited, though in a different context, i.e., when the biochemical coherence of the results is discussed.

### Reviewer 3: Fyodor Kondrashov, Center for Genomic Regulation, Spain

This is a quaint paper that focuses on providing a maximum likelihood approach for the reconstruction of gene content in LUCA. The nature of the study is methodological in nature such that the authors focus more on providing a method rather than interpreting the results. This feature of the manuscript, while being an advantage in some cases, in my opinion is a weakness in this case. Following a well-written introduction the results of the application of the ML approach does not provide any biological insight on the gene composition of LUCA. Table 2, especially given the short-hand notation for COG function, fails to excite a biologically oriented reader. In principle, the strength of this paper would be the applicability of the methodology by other users to obtain the biological insight that I found lacking.

Authors’ response: We have tried to improve the legend for all figures and tables.

Otherwise, this paper appears to represent a step in the argument about the gene content of LUCA, which is of interest to those studying this subject.

## Competing interests

The authors declare that they have no competing interests.

## Authors’ contributions

LK, HL and AM conceived the project; LK, HL, BR and AM analyzed the data; LK, HL and AM wrote the manuscript. All authors read and approved the final manuscript.

## Authors’ information

AM: the views expressed in this article are those of the author in his personal capacity and do not necessarily represent the view of the NSF or the United States.
